# Imaging Neurochemistry and Brain Structure Tracks Clinical Decline and Mechanisms of ALS in Patients

**DOI:** 10.3389/fneur.2020.590573

**Published:** 2020-12-03

**Authors:** Ovidiu C. Andronesi, Katharine Nicholson, Kourosh Jafari-Khouzani, Wolfgang Bogner, Jing Wang, James Chan, Eric A. Macklin, Mark Levine-Weinberg, Christopher Breen, Michael A. Schwarzschild, Merit Cudkowicz, Bruce R. Rosen, Sabrina Paganoni, Eva-Maria Ratai

**Affiliations:** ^1^Department of Radiology, A. A. Martinos Center for Biomedical Imaging, Harvard Medical School, Massachusetts General Hospital, Boston, MA, United States; ^2^Neurological Clinical Research Institute (NCRI), Massachusetts General Hospital, Boston, MA, United States; ^3^High Field MR Centre, Department of Biomedical Imaging and Image-Guided Therapy, Medical University of Vienna, Vienna, Austria; ^4^Department of Radiology, Union Hospital, Tongji Medical College, Huazhong University of Science and Technology, Wuhan, China; ^5^Biostatistics Center, Massachusetts General Hospital, Boston, MA, United States; ^6^MassGeneral Institute for Neurodegenerative Disease, Charlestown, MA, United States; ^7^Spaulding Rehabilitation Hospital, Boston, MA, United States

**Keywords:** magnetic resonance spectroscopic imaging (MRSI), neurochemistry, glutathione (GSH), neurodegeneration, T1 relaxation in the rotating frame (T1rho), macromolecular fraction, diffusion tensor imaging (DTI), amyotrophic lateral sclerosis (ALS)

## Abstract

**Background:** Oxidative stress and protein aggregation are key mechanisms in amyotrophic lateral sclerosis (ALS) disease. Reduced glutathione (GSH) is the most important intracellular antioxidant that protects neurons from reactive oxygen species. We hypothesized that levels of GSH measured by MR spectroscopic imaging (MRSI) in the motor cortex and corticospinal tract are linked to clinical trajectory of ALS patients.

**Objectives:** Investigate the value of GSH imaging to probe clinical decline of ALS patients in combination with other neurochemical and structural parameters.

**Methods:** Twenty-four ALS patients were imaged at 3 T with an advanced MR protocol. Mapping GSH levels in the brain is challenging, and for this purpose, we used an optimized spectral-edited 3D MRSI sequence with real-time motion and field correction to image glutathione and other brain metabolites. In addition, our imaging protocol included (i) an adiabatic T1ρ sequence to image macromolecular fraction of brain parenchyma, (ii) diffusion tensor imaging (DTI) for white matter tractography, and (iii) high-resolution anatomical imaging.

**Results:** We found GSH in motor cortex (*r* = −0.431, *p* = 0.04) and corticospinal tract (*r* = −0.497, *p* = 0.016) inversely correlated with time between diagnosis and imaging. N-Acetyl-aspartate (NAA) in motor cortex inversely correlated (*r* = −0.416, *p* = 0.049), while mean water diffusivity (*r* = 0.437, *p* = 0.033) and T1ρ (*r* = 0.482, *p* = 0.019) positively correlated with disease progression measured by imputed change in revised ALS Functional Rating Scale. There is more decrease in the motor cortex than in the white matter for GSH compared to NAA, glutamate, and glutamine.

**Conclusions:** Our study suggests that a panel of biochemical and structural imaging biomarkers defines a brain endophenotype, which can be used to time biological events and clinical progression in ALS patients. Such a quantitative brain endophenotype may stratify ALS patients into more homogeneous groups for therapeutic interventions compared to clinical criteria.

## Introduction

Neurodegeneration and neuroinflammation are the two major mechanisms in amyotrophic lateral sclerosis (ALS). The pathology of ALS begins with motor-neuron axonal dysfunction and retraction (1), which progresses retrogradely to the cell body death in motor cortex (2). In addition to motor-neuron degeneration due to protein aggregation (3–5), there is a marked neuroinflammatory reaction with astrocytic proliferation and microglial activation (6). The exact biological onset of ALS in patients is unknown but precedes the clinical onset of symptoms and signs. The dynamics of biological events is uncertain, heterogeneous, and variable in human ALS, yet it determines the clinical trajectory of disease progression in patients.

Oxidative stress (7) has been implicated as a key factor in the mechanisms of ALS disease. Reduced glutathione (GSH) is the major antioxidant used by mammalian cells to reduce reactive oxidative species (ROS), and the imbalance between the reductive capacity of the brain tissue and the production of reactive oxygen species results in a cellular environment that denaturates proteins (8), DNA, RNA, and unsaturated fatty acids. Additionally, there is a coupling between oxidative stress and other disease mechanisms such as glutamate excitotoxicity (9) through increased intracellular free calcium concentrations.

Aggregation of misfolded proteins caused by mutations, oxidative damage, or seeding (1, 10) leads to neurodegeneration in ALS. These protein aggregates disrupt cytoskeletal architecture, axonal trafficking, and mitochondrial functions. The prime examples of ALS proteinopathy are inclusions formed by mutated superoxide dismutase 1 (SOD1) (3, 4) and ubiquitylated/phosphorylated TAR DNA-binding protein 43 (TDP43) (5) that cannot be cleared by proteasomal and autophagic degradation machinery.

Currently, there is no effective therapy against ALS and the median patient survival is about 3 years from onset due to the progressive loss of vital motor functions such as respiration. It is expected that imaging biomarkers (11–14) that can probe biological mechanisms and timing of ALS pathology would be highly important and relevant for the success of medical interventions with disease-modifying treatments.

In this study, we performed multimodal quantitative neuroimaging using MR methods to probe the neurochemistry and brain structure in people with ALS disease. GSH measurement by MRS is challenging and requires special editing sequences to remove the large signals of overlapping metabolites (15, 16). For this purpose, we used a recently developed method (16, 17) that allows 3D imaging of GSH with more spatial coverage and at higher resolution than shown before in ALS patients by single voxel spectroscopy (18, 19). Hence, we were able to measure the GSH levels not only in the motor cortex of ALS patients as in previous studies (18, 19) but also in the corticospinal tract, which has not been investigated yet. Our method imaged GSH bilaterally in ALS patients, while previously, only one hemisphere was investigated. Restricting imaging to only one hemisphere is problematic in the case of ALS where symptoms may begin on one side and then progress to the other side.

We evaluated and compared how imaging biomarkers correlated with clinical variables related to ALS disease. First, we investigated whether GSH levels in the corticospinal tract may correlate stronger with disease duration than GHS levels in the motor cortex. Second, we investigated the relation between clinical ALS progression and other neuro-metabolites (20, 21) that are markers for neuronal health [N-acetyl-aspartate (NAA)], inflammation [myo-inositol (Ins)], and excitotoxicity [glutamate (Glu)]. Third, we investigated brain structural parameters (12, 14), such as cortical thickness, from anatomical MRI and white matter tractography (22) from diffusion tensor imaging (DTI). To probe brain parenchyma, we imaged tissue macromolecular fraction using longitudinal relaxation in the rotating frame (T1ρ) (23), which has not been previously studied in ALS patients. T1ρ measures molecular dynamics of water molecules interacting with tissue solid matrix. Hence, T1ρ might be sensitive to the change in the macromolecular tissue content due to accumulation and aggregation of misfolded proteins during the neurodegenerative process (24). Our objective was to assemble an extended panel of complementary quantitative imaging biomarkers that might help stratify ALS patients based on their underlying biology into more homogeneous groups for therapeutic interventions in addition to clinical scores such as the ALS Functional Rating Scale revised (ALSFRS-R).

## Materials and Methods

### Study Population

Human subjects were part of a pilot clinical trial that enrolled participants at Massachusetts General Hospital (MGH). The brain scans described here were performed at the baseline visit, prior to administration of the study drug. The Partners Human Research Committee approved this study. The trial was registered on clinicaltrials.gov (NCT02288091). Eligible participants had a diagnosis of possible, probable laboratory-supported, probable, or definite ALS by El Escorial criteria (25). Participants signed an informed consent form at screening. Medical history, detailed ALS history, physical and neurological examinations, medication review, vital signs, and laboratory tests were performed to determine eligibility as described in the clinical trial protocol. Clinical measurements included safety labs, slow vital capacity (SVC), and the ALS Functional Rating Scale–Revised (ALSFRS-R) questionnaire. There were no restrictions in vital capacity, disease duration, or riluzole use. However, the participants enrolled in our ALS study required for eligibility to have lower serum urate levels, which potentially may enrich for more rapid progressors (26, 27). Participants had to be willing and able to participate in brain magnetic resonance imaging (MRI) and magnetic resonance spectroscopic imaging (MRSI).

### Imaging

All MRI and MRSI measurements were performed using a clinical 3-T MR scanner (Tim Trio, Siemens, Erlangen) equipped with a 32-channel phased receive array head coil and a gradient system capable of 40 mT/m amplitude and 200 mT/m/s slew rate. Our imaging protocol had a total duration of 32 min, including anatomical, spectroscopic, T1ρ, and diffusion tensor DTI imaging.

### MRSI

A novel 3D MRSI sequence for GSH editing was designed using real-time motion correction and dynamic shimming, MEGA spectral editing, LASER adiabatic localization, and fast spiral spectroscopic imaging (17), which has been used recently for GSH mapping in patients with primary brain tumors (16). Acquisition parameters were repetition time (TR) = 1,600 ms, echo time (TE) = 68 ms, field of view (FOV) = 200 × 200 × 172 mm^3^, volume of interest (VOI) = 100 × 80 × 60 mm^3^, 14.3 mm isotropic voxels, acquisition matrix of 14 × 14 × 12 zero-filled to 16 × 16 × 16, *NA* = 10, and acquisition time of 12:05 min:s. J-difference MEGA editing was used to selectively edit GSH signal. The timing of the MEGA-LASER excitation was optimized for the maximum GSH signal for an echo time of 68 ms using MEGA Gaussian pulses of 60 Hz bandwidth applied in an interleaved fashion at 4.57 ppm (ON) and at −10 ppm (OFF) to edit the GSH signal at 2.95 ppm in the difference (ON – OFF) spectrum. LASER localization used adiabatic GOIA-W(16,4) pulses of 3.5 ms duration and 20 kHz bandwidth (28), which have a minimal chemical shift displacement error of 0.6% for 1 ppm. Shimming of the imaged brain volume (480 cm^3^) has been performed with a field mapping sequence for a global linewidth of the water of <15 Hz over the entire volume of interest.

3D MRSI data were fitted with LCModel (29) software to quantify the levels of GSH, NAA, glutamate and glutamine (Glx), Ins, and creatine (Cr). Cramer–Rao lower bounds (CRLB) <25% as calculated by LCModel were considered acceptable for metabolic goodness of fit. 3D metabolic maps were reconstructed from the LCModel fits using a combination of Montreal Neurological Institute (MINC), FSL (FMRIB Software Library, Oxford, UK), and MATLAB (Mathworks, Natick, MA) software tools, as detailed in (17). The GSH, NAA, Glx, and Ins signals were normalized relative to the level of total creatine (tCr = Cr + PCr). Metabolic maps were further coregistered to the MEMPRAGE T1-weighted anatomical image using robust register tools of Freesurfer software (Martinos Center, MGH) (30). Brain cortical gray matter segmentation obtained from Freesurfer and white matter tracts obtained with TRACULA (31) were used to calculate mean values of GSH/tCr, NAA/tCr, Glx/tCr, and Ins/tCr in anatomical regions of interests such as motor cortex and corticospinal tracts.

### T1ρ Imaging

The 3D T1ρ image acquisition was performed with a sequence that used turbo FLASH readout preceded by an adiabatic magnetization preparation module for longitudinal relaxation in the rotating frame (23). Low power gradient modulated slice-selective GOIA-W(16,4) adiabatic inversion pulses of 4 ms and 5 kHz bandwidth were concatenated in a pulse train with no gaps according to the MLEV-4 phase cycle scheme (32) to obtain longitudinal magnetization preparation times of 0, 16, 32, 48, and 64 ms. The following acquisition parameters were used: FOV = 256 × 256 × 170 mm^3^, matrix = 192 × 192 × 128, voxel size = 1.33 × 1.33 × 1.33 mm^3^, partial Fourier factor of 6/8 in the slice and phase encoding directions, parallel acquisition (PAT) factor 2, readout bandwidth = 1,530 Hz/pixel, flip angle = 8°, TE = 0.98 ms, TR = 0.8 s, 10 dummy scans, and five preparation times (0, 16, 32, 48, 64 ms). The total acquisition time was 5:40 min.

Relaxation decay curves were fitted voxel-wise with a mono-exponential *S*_1ρ_(*t*) = *S*(0)exp(−*t*/*T*_1ρ_) equation to obtain maps of T1ρ relaxation times. Parametric maps for the fraction of water bound to macromolecules (WBMs) were obtained by voxel-wise non-linear least square fitting of a mathematical model for two site exchange in the fast limit (2SX/FXL) between a pool of free water and a pool of water bound to macromolecules (23). Motion correction was performed retrospectively by aligning the volumes for each preparation time. Only voxels with goodness of fit by coefficient of determination R^2^ > 0.95 were included in the statistical analysis.

### Diffusion Tensor Imaging and Analysis

Diffusion tensor imaging (DTI) was acquired with echo planar imaging (EPI) employing a high b value of 1,000 s/mm^2^ and 20 gradient directions with 1.9 mm isotropic voxels (TR/TE = 7,980/84 ms). Real-time motion correction was performed during DTI acquisition with an interleaved navigator (33). To correct EPI distortions due to diffusion gradient eddy currents, two additional image acquisitions were made with opposing polarities of the phase-encode blips. Diffusion-weighted images were processed with FSL software, including distortion correction with TOPUP method (34). Eddy current and additional motion artifacts were also corrected using eddy_correct tool in FSL. We used AFNI tools (35) to update the gradient tables (reorient b-vectors) after artifact correction. Fractional anisotropy (FA) and mean diffusivity (MD) were then generated by FDT in FSL. Tract-specific statistics for fractional anisotropy, mean diffusivity, axial diffusivity (AD), and radial diffusivity (RD) in 18 major white matter tracts, including the corticospinal tract, were obtained using TRACULA (31). For cortical gray matter of motor cortex, only FA and MD were analyzed, since AD and RD measures are characteristic for white matter tract structures.

### Brain Segmentation and Volumetrics

T1-weighted anatomical image of the brain were acquired using multiecho MPRAGE (MEMPRAGE) sequence (36) with 1 mm isotropic resolution and the following acquisition parameters (inversion/repetition/echo times, TI/TR/TE) TI/TR/TE1/TE2/TE3/TE4/ = 1,200/2,530/1.64/3.5/5.36/7.22 ms, 1 mm isotropic, 256 × 256 × 176 matrix, and 5:56 acquisition time. The Freesurfer software (37) was used for segmentation of the MEMPRAGE images to obtain volumes and cortical thickness of relevant brain regions.

### Statistical Analysis

The relationship between imaging biomarkers and clinical variables was investigated using Pearson correlation for continuous variables and one-way analysis of variance (ANOVA) for categorical variables. All tests were two-sided using an alpha of 0.05. Clinical variables included age, months from symptom onset to diagnosis (diagnostic delay), months from symptom onset to baseline (disease duration), months from diagnosis to MRI scan, ALSFRS-R total score, rate of disease progression/month as measured by the imputed ALSFRS-R rate (calculated by subtracting the current ALSFRS-R total score from 48 and then dividing by the number of months since symptom onset), slow vital capacity (SVC), gender, and site of onset. Analyses were carried out in R software, version 3.4.3 (R: A language and environment for statistical computing. R Foundation for Statistical Computing, Vienna, Austria. URL https://www.R-project.org/).

### Data Availability

The datasets generated during and/or analyzed during the current study are available from the corresponding author on reasonable request.

## Results

The clinical and demographic distribution of the 24 ALS participants included in our study are listed in Table 1 given as percentage for categorical variables and mean ± SD for continuous variables. Briefly, the subjects' distribution at the time of the brain scan was as follows: (1) 25% male, (2) 62.5% limb onset, (3) age of 61 ± 8 years, (4) diagnostic delay of 12 ± 7 months, (5) time between diagnosis and MRI scan of 16 ± 15 months, (6) total disease duration until the MRI scan 28 ± 17 months, (7) ALSFRS-R score 35.7 ± 5.4 months, (8) rate of disease progression by the imputed change of ALSFRS-R total score of 0.64 ± 0.44 decline/month, and (9) slow vital capacity (SVC) 86% ± 31.

**Table 1:** Clinical and demographic distribution of the study cohort (*N* = 24, amyotrophic lateral sclerosis (ALS) human subjects).

**Clinical variable**	**Percent (n) or mean (SD)**
Age	60.8 (8.32) years
Time from symptom onset to diagnostic (Sx–Dx)	12.1 (6.51) months
Time from diagnostic to MRI scan (Dx–MRI)	15.6 (15.09) months
Time from symptom onset to MRI scan (Sx–MRI)	27.7 (17.22) months
ALSFRS-R	35.7 (5.38)
Rate of disease progression	0.6 (0.44) months^−1^
SVC	86.1 (31.10)
**Gender**	
Male	25.0% (6)
Female	75.0% (18)
**Site onset**	
Limb	62.5% (15)
Bulbar	37.5% (9)

*Percent and number of participants (n) are shown for categorial variables; mean and standard deviation (SD) are shown for continuous variables. Rate of disease progression is presented as monthly change in ALSFRS-R total score (points/month). SVC is presented as percent of predicted values. Individual values for all patients are given in Supplementary Table 1*.

*ALSFRS-R, ALS functional rating scale revised; SVC, slow vital capacity; Sx, symptom onset; Dx, diagnostic onset*.

**Figure 1:**
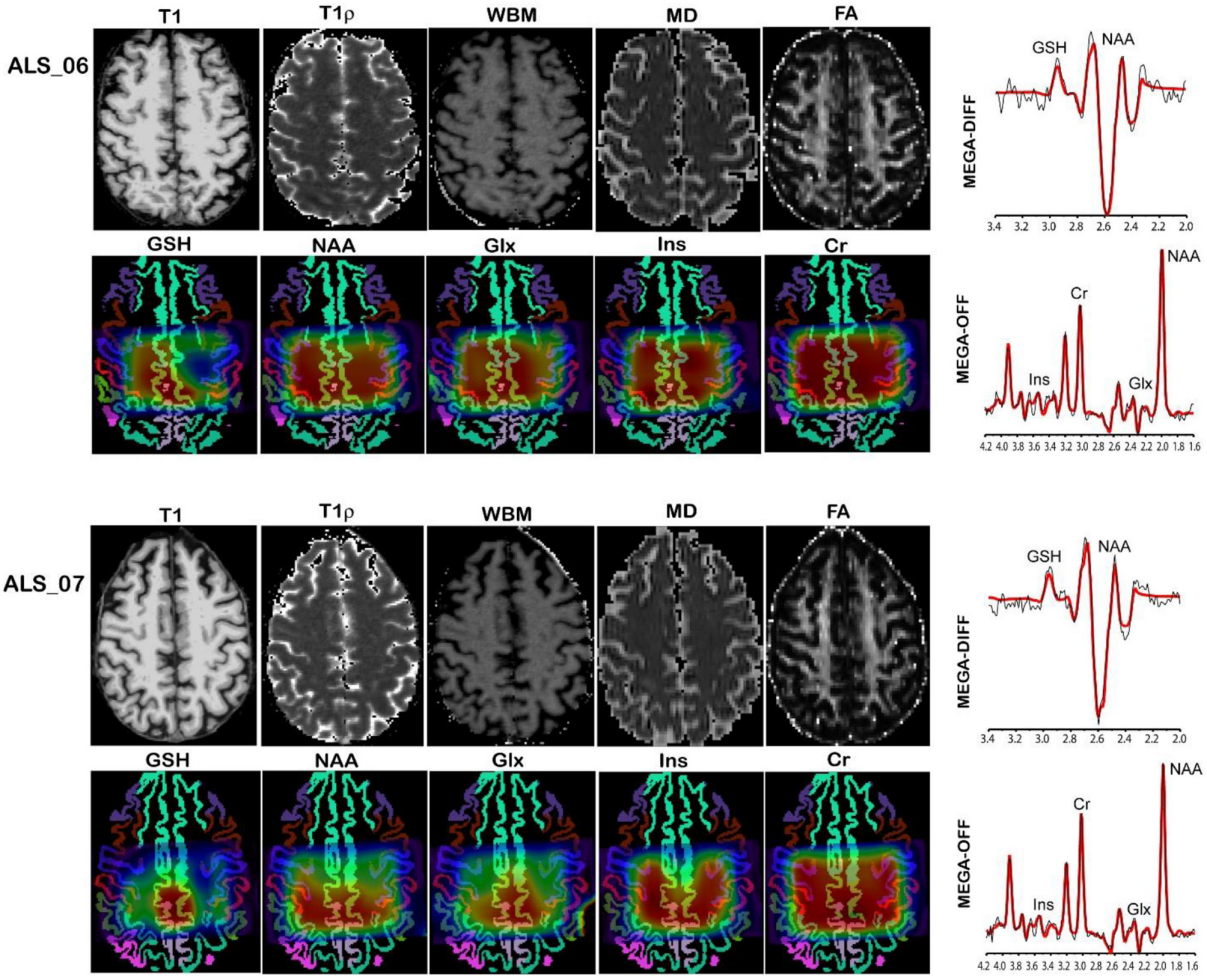
MR imaging and spectroscopy in two amyotrophic lateral sclerosis (ALS) participants with high ALS Functional Rating Scale revised (ALSFRS-R) total score (upper panel) and low ALSFRS-R total score (lower panel). Metabolic maps are shown overlaid on gray matter brain segmentation. Levels of glutathione (GSH), N-acetyl-aspartate (NAA), and glutamate and glutamine (Glx) are visibly lower in the motor cortex (blue ribbon segmentation) compared to other brain regions. Examples of difference (MEGA-DIFF) and off (MEGA-OFF) spectra show good signal-to-noise ratio for GSH, NAA, Glx, myo-inositol (Ins), and creatine (Cr). Spectral fitting by LCModel (red line) is superimposed on the experimental data (black line).

**Figure 2:**
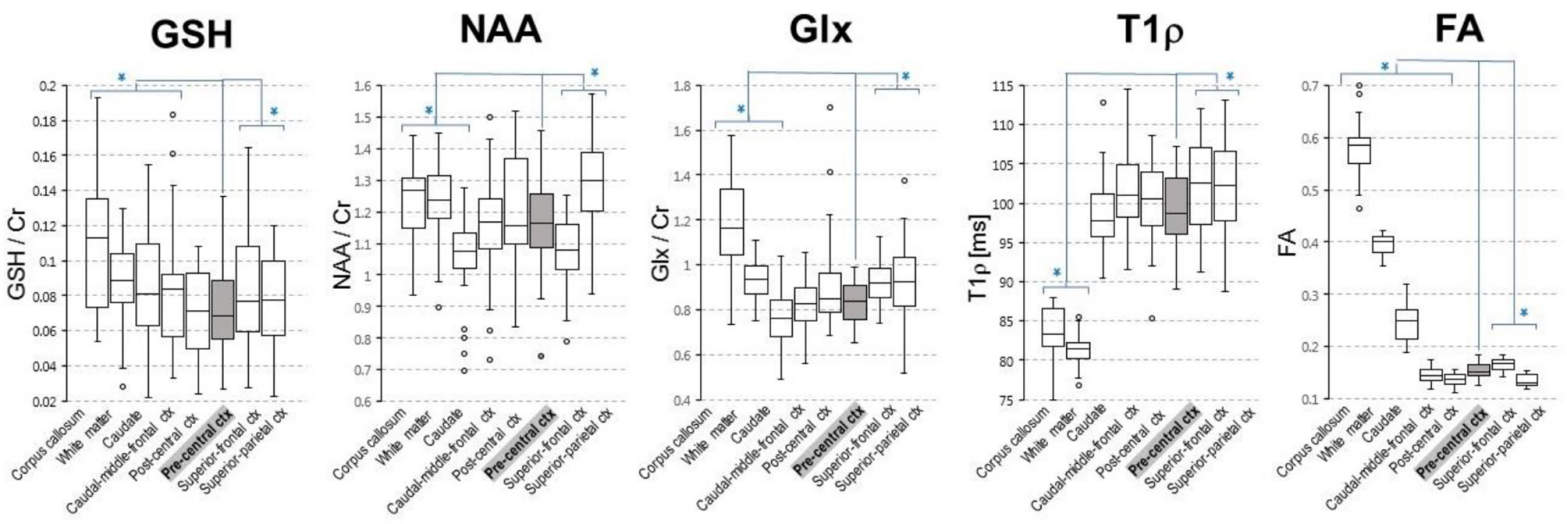
Group level regional differences for brain metabolites [glutathione (GSH), N-acetyl-aspartate (NAA), and glutamate and glutamine (Glx)] and T1ρ and fractional anisotropy. Values for both right and left hemisphere are shown for each imaging parameter. Eight cortical and subcortical brain regions that are consistently included in the MRSI volume across all volunteers are shown. Asterisks indicates regions that are statistically different (*p* < 0.05) from the motor cortex (pre-central ctx, highlighted in gray).

**Figure 3:**
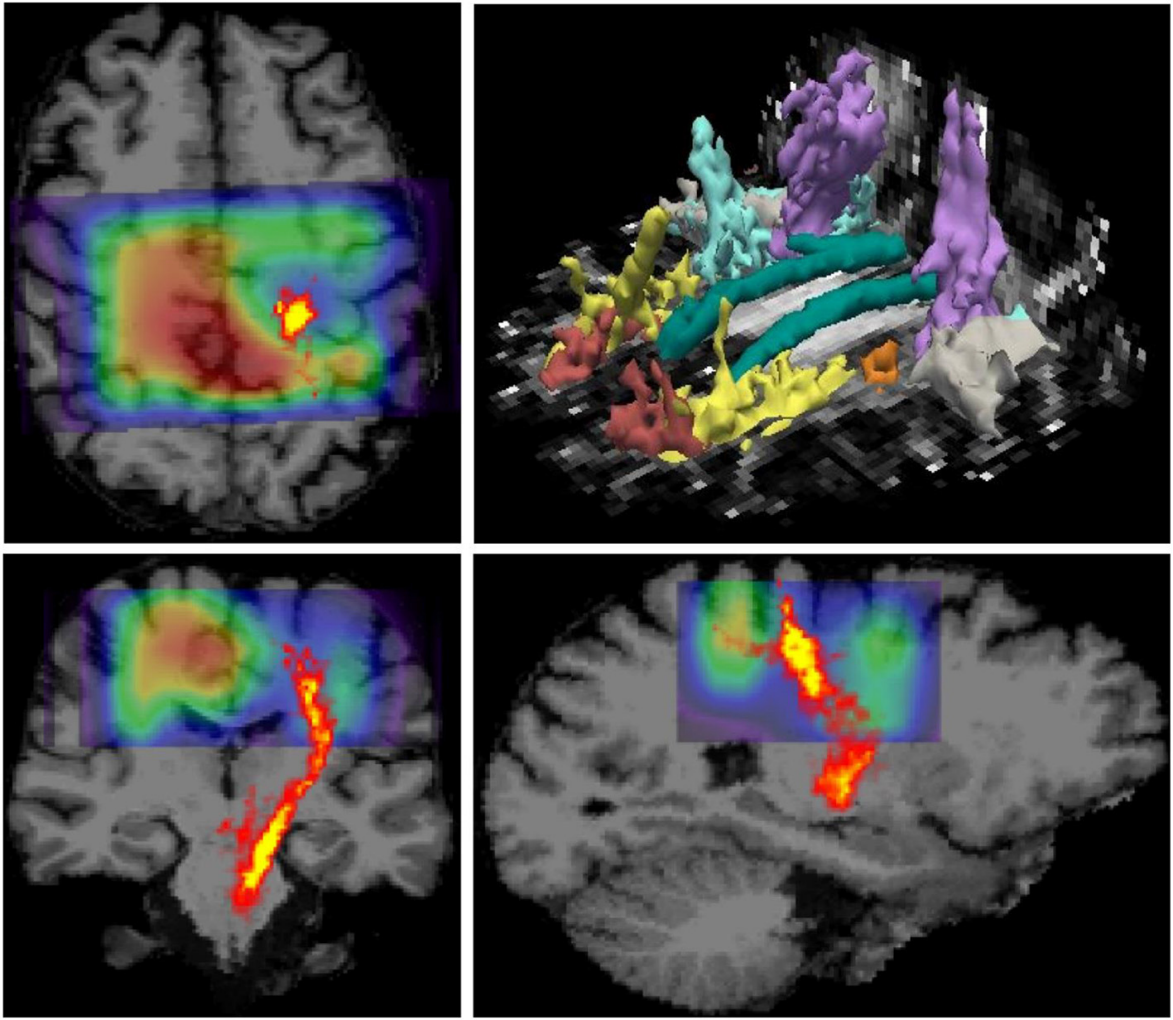
Overlay between glutathione (GSH) map derived from 3D MR spectroscopic imaging (MRSI) and the corticospinal tract pathway derived from diffusion tensor imaging (DTI) is shown on top of the anatomical MRI scan. The data correspond to the patient shown in the upper panel in Figure 1, which has pronounced weakness on the side contralateral to the hemisphere with reduced GSH in the motor area. It can be noted that GSH is reduced along the corticospinal tract (CST) pathway, in addition to the GSH reduction in the motor cortex. The image in the right upper corner shows major white matter tracts reconstructed by TRACULA.

MR imaging and spectroscopic data are shown in Figure 1 for a subject with a high ALSFRS-R score (upper panel) and a subject with a low ALSFRS-R score (lower panel), both with limb onset. Examples of difference and OFF spectra show good signal to noise for the edited GSH signal and the other neurometabolites that were analyzed. Metabolic maps for GSH, NAA, and Glx indicate lower levels in the motor cortex (MC) compared to other brain regions, which are apparent at single subject level as visible for both subjects shown in Figure 1. In the participant with higher ALSFRS score (Figure 1, upper panel) the decrease in GSH, NAA, and Glx involves especially the motor cortex contralateral to the most affected side, and there is more decrease for GSH compared to NAA and Glx. By contrast, in the participant with lower ALSFRS score (Figure 1, lower panel), there is comparable bilateral decrease in neurometabolites (GSH, NAA, and Glx) in both motor cortexes, which matches the clinical presentation with bilateral weakness. Of notice, the decrease in motor cortex relative to other brain regions is more pronounced for GSH compared to NAA or other metabolites. The map of Cr is largely uniform over the imaged volume, which suggests that Cr might be used as a reference signal for metabolic ratios in ALS. The anatomical images, the maps of T1ρ relaxation times, water bound to macromolecules, mean diffusivity, and fractional anisotropy show less difference between motor cortex and other brain areas at the individual subject level shown in Figure 1, but significant effects were found at group level by statistical analysis. Figure 2 shows brain regional differences at group level for glutathione, NAA, glutamate, and glutamine, T1ρ, and fractional anisotropy. Statistical significant differences are indicated between the motor cortex and other seven cortical and subcortical areas that were consistently included and common between MRSI and MRI volumes. Across these brain regions, the GSH has the lowest value in the motor cortex, while for the other imaging parameters, the distribution has a different shape. In particular, there is a significant decrease in GSH in the motor cortex by 21% (p < 0.05) compared to the white matter, while the NAA and Glx in the motor cortex are decreased by 6 and 13% (p < 0.05), respectively, compared to the white matter. Compared to the white matter, the motor cortex values for T1ρ are 18% higher (p < 0.05), water-bound fraction is 14% lower (p < 0.05), and fractional anisotropy is 2.5 times lower (p < 0.05). In Figure 3, the overlay between the GSH map and the corticospinal tract (CST) is shown for the first participant, where it is readily visible that GSH is decreased all along the CST pathway. A summary with quantitative analysis of imaging parameters for the motor cortex is presented in Table 2 and for the corticospinal tract in Table 3.

**Table 2:** Imaging biomarker values in the motor cortex (average of right and left hemispheres) of the 24 amyotrophic lateral sclerosis (ALS) subjects.

	**GSH/Cr**	**NAA/Cr**	**Glx/Cr**	**Ins/Cr**	**T1ρ (ms)**	**WBM (%)**	**MD (mm^**2**^/ms)**	**FA (0–1)**	**Thickness (mm)**
**Mean**	0.071	1.22	0.83	0.76	98.7	9.23	1.11	0.15	2.36
**SD**	0.021	0.12	0.07	0.11	5.6	0.49	0.08	0.02	0.16

*Individual values for all participants are given in Supplementary Table 2*.

**Table 3:** Imaging biomarker values in the corticospinal tract (average of right and left hemispheres) of the 24 amyotrophic lateral sclerosis (ALS) subjects.

	**GSH/Cr**	**NAA/Cr**	**Glx/Cr**	**Ins/Cr**	**T1ρ (ms)**	**WBM (%)**	**AD (mm^**2**^/ms)**	**RD (mm^**2**^/ms)**	**MD (mm^**2**^/ms)**	**FA (0–1)**	**Volume (mm^**3**^)**
Mean	0.087	1.46	0.91	0.87	78.2	10.78	1.31	0.53	0.79	0.55	1,952
SD	0.023	0.15	0.08	0.15	2.5	0.28	0.05	0.05	0.04	0.04	438

*Individual values for all participants are given in Supplementary Table 3*.

*GSH, glutathione; NAA, N-acetyl-aspartate; Glx, glutamate and glutamine; Ins, myo-inositol; Cr, creatine; T1r, longitudinal relaxation in the rotating frame; WBM, water bound to macromolecules; AD, axial diffusivity; RD, radial diffusivity; MD, mean diffusivity; FA, fractional anisotropy*.

**Figure 4:**
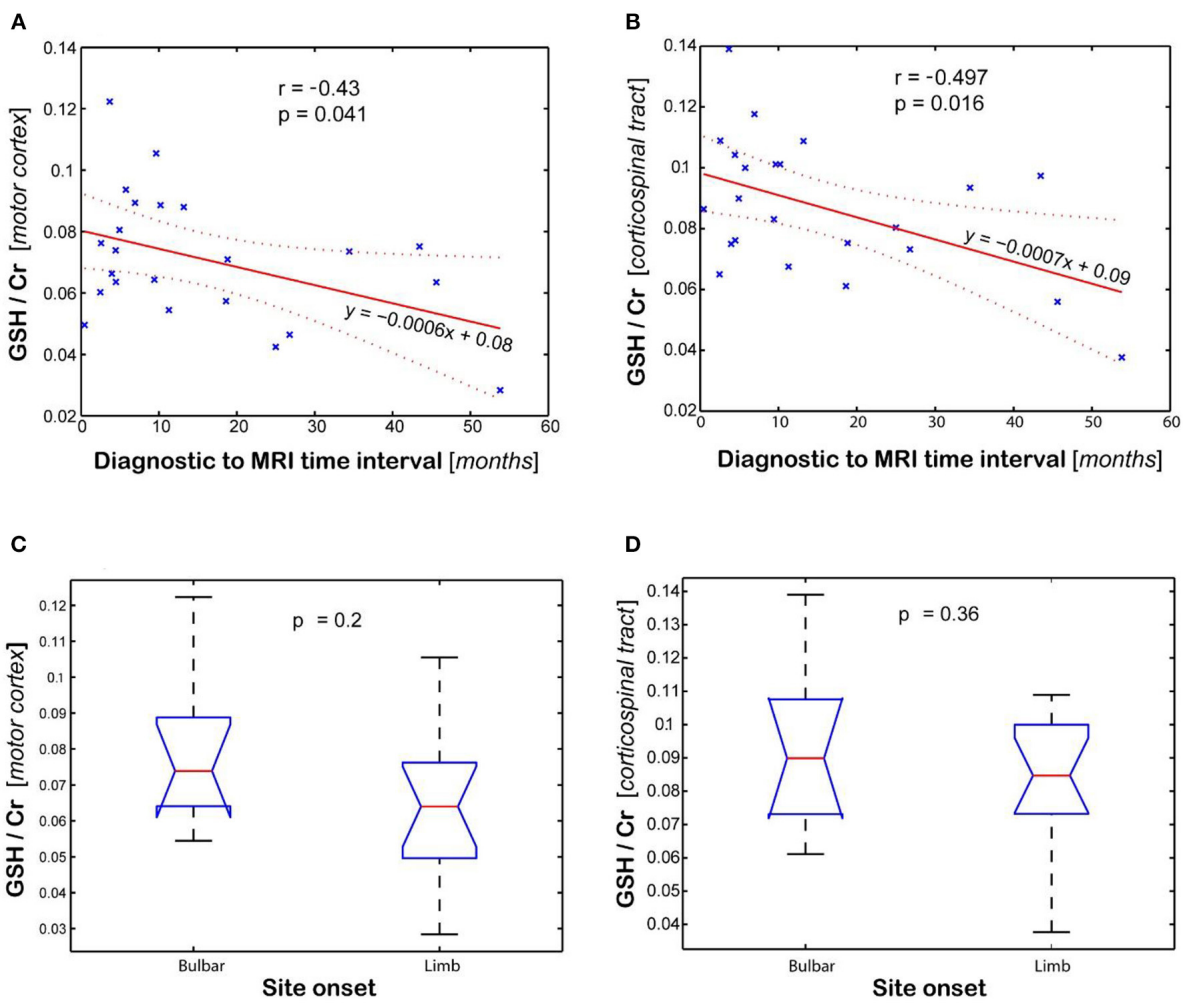
Association between glutathione (GSH) levels (GSH/Cr) and clinical variables (diagnostic to MRI time interval and site onset). Plots show the linear regression model for time interval and ANOVA analysis for site onset in the **(A,C)** motor cortex and **(B,D)** corticospinal tract, respectively. Values are average of the left and right brain regions.

Statistical analysis of GSH levels is shown in Figure 4 for the motor cortex (Figures 4A,C) and corticospinal tract (Figures 4B,D). The GSH/Cr ratio in motor cortex inversely correlates (*r* = −0.43, *p* = 0.04, Figure 4A) with time interval between diagnosis and MRI scan, while it shows only a trend for inverse correlation with total disease duration (time between symptom onset and MRI, *r* = −0.401, *p* = 0.058). A stronger inverse correlation (*r* = −0.497, *p* = 0.016, Figure 4B) is found between the GSH/Cr ratio in CST and time interval of diagnosis to MRI, with only a trend for inverse correlation with total disease duration (*r* = −0.407, *p* = 0.054). There is no clear separation for GSH/Cr ratio in corticospinal tract based on site onset (*p* = 0.36, Figure 4D). There is no statistical correlation between the GSH/Cr ratio and the ALSFRS-R score at the time of the scan.

**Figure 5:**
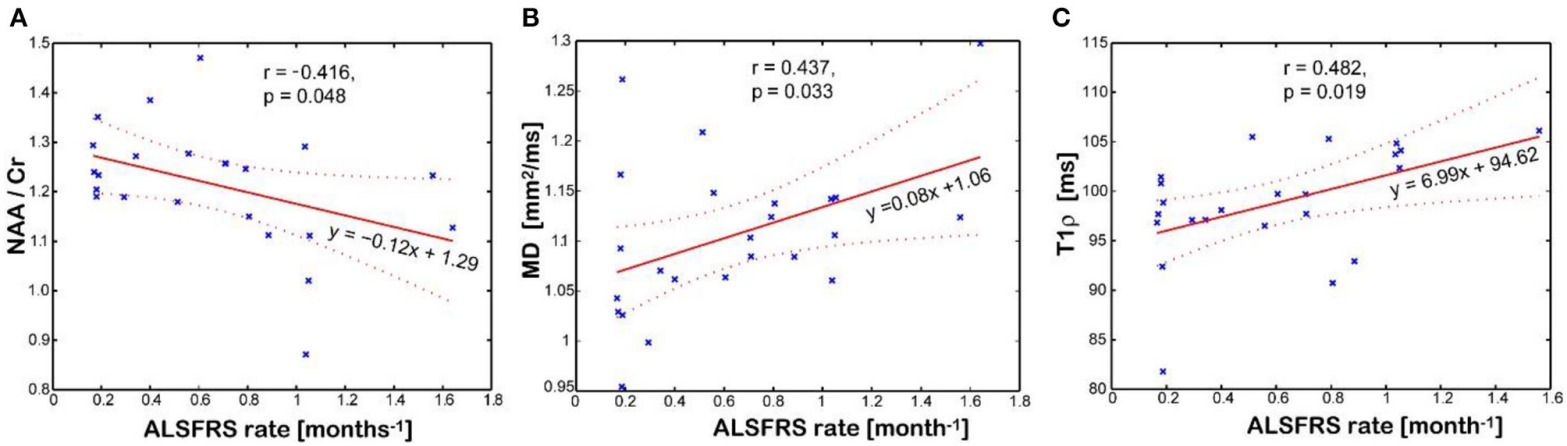
Association between disease progression measured by the imputed ALSFRS rate and the imaging biomarkers measured in the motor cortex: **(A)** N-acetyl-aspartate (NAA)/creatine (Cr) levels; **(B)** mean diffusivity (MD); **(C)** and T1rho relaxation time in the rotating frame. Values of imaging parameters are average of left and right regions.

Figure 5 shows imaging biomarkers that correlated with the rate of clinical progression measured by the imputed ALSFRS-R rate. The NAA/Cr ratio inversely correlates (*r* = −0.416, *p* = 0.048, Figure 5A), while mean water diffusivity (*r* = 0.437, *p* = 0.033, Figure 5B) and T1ρ relaxation (*r* = 0.482, *p* = 0.019, Figure 5C) directly correlate with the rate of disease progression, respectively. Interestingly, the T1ρ relaxation has the strongest and statistically most significant correlation with the imputed ALSFRS-R rate. Cortical thickness of the motor cortex had no correlation with any clinical parameter. There was no association between Glx and Ins with clinical variables.

## Discussion

Our study shows that 3D mapping of brain metabolism, including difficult to measure metabolites such as glutathione, can be feasibly performed in ALS patients within a clinically acceptable scan time. The total duration of our scan protocol (32 min), which included MRSI, T1ρ, DTI, and anatomical imaging, was well-tolerated by all the ALS participants in this cohort. The brain coverage of our GSH imaging was larger (bilateral) with higher resolution than previous ALS studies (18, 19). Hence, we measured GSH levels not only in the motor cortex as in previous studies but simultaneously also in the corticospinal tracts, which may help probe the direction of ALS disease progression. Editing methods based on spectral subtraction are sensitive to motion (38), and to improve robustness of GSH imaging, we employed real-time motion correction and shim update (16, 17). This is particularly important for ALS patients, which, due to their condition, might have involuntary head movements during imaging.

Our results suggest that GSH and NAA provide complementary neurochemical information on the glial and neuronal brain compartments, respectively. GSH is the most abundant antioxidant molecule in the brain (39, 40). Astrocytes are the major source of GSH in the brain, and the shuttling of GSH precursors from astrocytes to neurons appears critical in the neuroprotective effects of astrocytes against neurodegeneration (41–44), at least in culture and animal models. Thus, GSH measurements *in vivo* may reflect astrocyte biology and glial activation during disease progression. We found that GSH levels in the motor cortex and corticospinal tract inversely correlated with time from diagnosis to imaging. The GSH levels in the corticospinal tract had a stronger correlation with disease duration than motor cortex values, which has not been shown to date. Hence, the decrease in GSH levels in the corticospinal tract might be an earlier biomarker for the onset of ALS disease compared to GSH levels in the motor cortex. This result may suggest a retrograde gradient of motor neuron degeneration in our cohort of ALS patients. Other studies measuring NAA levels found reduced levels of NAA/Cr in precentral gyrus and corona radiata but normal levels in the internal capsule and cerebral peduncle when comparing ALS patients to normal subjects (45). However, in our study, GSH did not correlate with measures of disease severity (i.e., ALSFRS-R total score at the time of the scan) or rate of disease progression (i.e., monthly decline in ALSFRS-R from onset to the time of the scan). This may be explained if involvement of lower motor neuron is more significant than upper motor neuron probed by our GSH measurements or by the sample size. In addition, two recent single voxel spectroscopy studies (18, 19) investigated the GSH levels in the motor cortex of people with ALS disease and found also no correlation with the ALSFRS-R score or with total disease duration from symptom onset, similarly to our findings. However, in our study, GSH correlated with time between diagnosis and the MR scan. The fact that GSH correlated with time from diagnosis but not with time from symptoms onset might be explained by the fact that ALS onset is insidious and the exact timing of it may be biased by patient recollection, while the time of diagnosis can be reliably ascertained from medical records. In contrast to GSH, the levels of NAA in the motor cortex correlated with the rate of disease progression, which supports the fact that NAA is a reliable neuronal marker as previously shown (18–21, 46–49) and can be used to probe the progressive neurodegeneration during ALS disease. Interestingly, the metabolic maps shown in the upper panel of Figure 1 indicate that there is more pronounced decrease in motor cortex for GSH than NAA at early stages of the disease, which have been found also at group level (Figure 2), suggesting that astrocytic changes might precede neurodegeneration or that the two processes may follow a different time course. Hence, the combination of GSH and NAA may probe the dynamics of both pathophysiological mechanisms involved in ALS, astrocytic activation, and neurodegeneration, respectively. The maps in Figure 3 from the same individual indicate that GSH is decreased along the CST tract. The CST tract has been found by DTI to be altered in ALS patients compared to healthy controls (22). Our results on GSH-DTI correlation add to the findings of previous studies (48, 50, 51) combining MRSI and DTI in ALS patients that have found association between changes in NAA and water diffusion in patients with motor neuron disease. We found that, at group level, there is stronger correlation with disease duration for GSH in the corticospinal tract than for GSH in the motor cortex (Figure 4B), which was not previously described and would corroborate with a retrograde gradient of degeneration from axon toward the cell body of neurons. Other metabolites such as Glx and Ins were not found in our study to correlate significantly with clinical variables, although previous studies (52) reported such results. The different results in our case may be explained by different severity of ALS disease in our group of patients compared to previous studies.

Our advanced MRSI protocol provided several advantages over single voxel methods that were previously used in ALS studies to measure GSH levels. In earlier studies methods (18, 19), spectra were recorded only from one side of the motor cortex, contralateral to the most affected site, with large voxels (12–20 cm^3^) and long acquisition times (>15 min). In ALS, symptoms may begin on one side and then progress to the other side; hence, it is desirable to investigate the brain bilaterally. Our MRSI protocol encompassed both motor cortexes and corticospinal tracts at higher spatial resolution and in a shorter acquisition time.

Anatomical and structural imaging reveal mixed results. Cortical thickness of the motor cortex had no association with any of the clinical parameters suggesting that cortical thinning due to marked neuronal loss might not be observed until later stages of disease progression compared to the stage of our ALS participants (12, 53). While cortical thickness does not seem to be a sensitive marker for the early stages of neurodegeneration and neuronal loss, other structural imaging parameters may probe changes due to the buildup of protein aggregation in the neuronal bodies. Specifically, longitudinal relaxation in the rotating frame (T1ρ) seems to show greater association with the disease stage compared to water diffusion. This may be explained by the fact that T1ρ probes water bound to macromolecules, while mean diffusivity or fractional anisotropy probe free water. The increased effect of neurodegeneration on the T1ρ results from the combination of two biophysical phenomena: on the one hand, the accumulation of misfolded protein aggregates increases the macromolecular load; on the other hand, misfolded proteins have large unstructured end terminal domains where backbone and residues are more accessible to water compared to well-structured folded proteins (54, 55). Both these biophysical mechanisms will lead to an increase in the number of water molecules that come in contact with the macromolecular environment, increasing the relaxation in the rotating frame. Due to technical limitations, T1ρ has not been used previously in ALS patients, but here, we were able to perform high-resolution whole-brain T1ρ imaging with mitigation of B_1_ field artifacts in a short acquisition time by a novel low power adiabatic T1ρ sequence (23).

Because our study did not include a matched healthy control group, we were not able to evaluate the diagnostic value of our imaging methods. Instead, our study was focused on probing disease severity that occurs only in patients. Our patient cohort was limited, which may explain differences in some results compared to previous ALS studies (13, 19, 21, 48, 49). Our results would also need to be confirmed in larger studies to better establish the value of proposed imaging methods for stratification and prognosis of ALS patients. However, our imaging study is one of the largest using MRSI for ALS patients, in particular to measure GSH levels, which was integrated in an advanced imaging protocol that was well-tolerated by all participants.

In summary, our study suggests that, by using advanced MR imaging, a disease endophenotype may be determined and quantified in ALS patients. Such an imaging endophenotype could be closer to the molecular mechanisms of ALS compared to the heterogeneous phenotype probed by clinical criteria. Hence, it could be applied to investigate presymptomatic gene carrier (56, 57) subjects predisposed to higher risk of developing ALS disease and to stratify patients (12, 58) into more homogeneous groups for clinical trials and therapeutic interventions.

## Data Availability Statement

The datasets generated during and/or analyzed during the current study are available from the corresponding author on reasonable request.

## Ethics Statement

The studies involving human participants were reviewed and approved by Partners Human Research Committee. The patients/participants provided their written informed consent to participate in this study. Written informed consent was obtained from the individual(s) for the publication of any potentially identifiable images or data included in this article.

## Author Contributions

OCA: developed imaging methods, imaged patients, analyzed data, study design, and wrote the manuscript. KJ-K: analyzed data and wrote the manuscript. WB: developed imaging methods and wrote manuscript. JW, ML-W, CB, and E-MR: imaged patients and wrote manuscript. JC and EM: statistical analysis and wrote manuscript. MS, MC, and BR: study design and wrote manuscript. SP: study design, analyzed data, and wrote manuscript. All authors contributed to the article and approved the submitted version.

## Conflict of Interest

The authors declare that the research was conducted in the absence of any commercial or financial relationships that could be construed as a potential conflict of interest.
