# Supplementary material for: Imaging Neurochemistry and Brain Structure Tracks Clinical Decline and Mechanisms of ALS in Patients
Source: Front Neurol. 2020 Dec 3;11:590573. doi: 10.3389/fneur.2020.590573 (PMC7744722; doi:10.3389/fneur.2020.590573)
Supplement: Supplementary file 1 [file Table_1.DOCX]

| Pt_ID | Age  [years] | Gender | Site onset | Sx-Dx  [months] | | Dx-MRI  [months] | | Sx-MRI  [months] | ALSFRS | ALSFRS rate [months^-1^] | SVC |
| --- | --- | --- | --- | --- | --- | --- | --- | --- | --- | --- | --- |
| ALS_01 | 65.09 | Female | Limb | 5.82 | 5.76 | | 11.58 | | 29 | 1.64 | 45.98 |
| ALS_02 | 54.05 | Female | Limb | 17.57 | 14.47 | | 32.04 | | 42 | 0.19 | 108.57 |
| ALS_03 | 67.97 | Female | Bulbar | 10.00 | 6.94 | | 16.94 | | 33 | 0.89 | 24.34 |
| ALS_04 | 54.42 | Female | Bulbar | 16.02 | 4.93 | | 20.95 | | 26 | 1.05 | *n.a.* |
| ALS_05 | 62.05 | Female | Bulbar | 12.99 | 10.20 | | 23.19 | | 24 | 1.03 | 20.83 |
| ALS_06 | 63.36 | Female | Limb | 23.03 | 13.19 | | 36.22 | | 42 | 0.17 | 94.56 |
| ALS_07 | 50.97 | Female | Limb | 5.03 | 9.41 | | 14.44 | | 33 | 1.04 | 95.17 |
| ALS_08 | 67.89 | Female | Limb | 16.02 | 43.45 | | 59.47 | | 37 | 0.18 | 102.21 |
| ALS_09 | 51.32 | Female | Limb | 24.05 | 34.44 | | 58.49 | | 37 | 0.19 | 101.85 |
| ALS_10 | 63.30 | Male | Limb | 6.41 | 2.57 | | 8.98 | | 34 | 1.56 | 121.19 |
| ALS_11 | 43.32 | Female | Limb | 4.44 | 45.63 | | 50.07 | | 39 | 0.18 | 59.03 |
| ALS_12 | 52.50 | Male | Bulbar | 8.16 | 18.62 | | 26.78 | | 29 | 0.71 | 70.96 |
| ALS_13 | 56.60 | Female | Bulbar | 20.07 | 18.82 | | 38.88 | | 41 | 0.18 | 96.31 |
| ALS_14 | 67.23 | Female | Bulbar | 12.01 | 11.28 | | 23.29 | | 35 | 0.56 | 54.01 |
| ALS_15 | 63.36 | Female | Limb | 5.86 | 0.46 | | 6.32 | | 43 | 0.79 | 97.23 |
| ALS_16 | 63.45 | Female | Bulbar | 8.78 | 3.95 | | 12.73 | | 39 | 0.71 | 89.33 |
| ALS_17 | 56.69 | Female | Bulbar | 9.97 | 3.68 | | 13.65 | | 37 | 0.81 | 85.37 |
| ALS_18 | 73.37 | Male | Limb | 15.99 | 4.47 | | 20.46 | | 42 | 0.29 | 82.66 |
| ALS_19 | 54.42 | Male | Limb | 4.01 | 9.64 | | 13.65 | | 41 | 0.51 | 116.73 |
| ALS_20 | 62.74 | Female | Limb | 4.97 | 53.82 | | 58.78 | | 38 | 0.17 | 126.54 |
| ALS_21 | 72.64 | Female | Limb | 25.00 | 25.00 | | 50.00 | | 28 | 0.40 | 49.88 |
| ALS_22 | 74.41 | Male | Limb | 14.21 | 26.78 | | 40.99 | | 34 | 0.34 | 137.62 |
| ALS_23 | 69.29 | Female | Bulbar | 6.94 | 4.44 | | 11.38 | | 36 | 1.05 | 102.98 |
| ALS_24 | 49.73 | Male | Limb | 14.05 | 2.47 | | 16.51 | | 38 | 0.61 | 97.67 |

**Supplementary Table 1.** Clinical and demographic data of the 24 ALS patients.

| \| **Pt_ID**  **[ALS_#]** \| **GSH**  **/ Cr** \| **NAA**  **/ Cr** \| **Glx**  **/ Cr** \| **Ins**  **/ Cr** \| **T1ρ [ms]** \| **WBM [%]** \| **MD [mm^2^/ms]** \| **FA**  **[0-1]** \| **Thickness [mm]** \| \| --- \| --- \| --- \| --- \| --- \| --- \| --- \| --- \| --- \| --- \| \| **ALS_01** \| 0.094 \| 1.127 \| 0.762 \| 0.592 \| *n.a.* \| *n.a.* \| 1.298 \| 0.103 \| 2.409 \| \| **ALS_02** \| *n.a.* \| *n.a.* \| *n.a.* \| *n.a.* \| 81.8 \| 10.74 \| 1.262 \| 0.132 \| 2.414 \| \| **ALS_03** \| 0.089 \| 1.112 \| 0.825 \| 0.833 \| 92.9 \| 9.41 \| 1.084 \| 0.151 \| 2.403 \| \| **ALS_04** \| 0.081 \| 1.020 \| 0.787 \| 0.948 \| 102.4 \| 10.07 \| 1.106 \| 0.187 \| 2.089 \| \| **ALS_05** \| 0.089 \| 1.291 \| 0.912 \| 0.960 \| 103.7 \| 8.84 \| 1.142 \| 0.161 \| 2.244 \| \| **ALS_06** \| 0.088 \| 1.294 \| 0.940 \| 0.872 \| 96.8 \| 9.24 \| 1.043 \| 0.180 \| 2.466 \| \| **ALS_07** \| 0.064 \| 0.871 \| 0.682 \| 0.916 \| 104.9 \| 8.87 \| 1.061 \| 0.139 \| 2.169 \| \| **ALS_08** \| 0.075 \| 1.351 \| 0.909 \| 0.727 \| 92.4 \| 9.78 \| 0.954 \| 0.163 \| 2.242 \| \| **ALS_09** \| 0.074 \| 1.234 \| 0.877 \| 0.795 \| 98.8 \| 9.09 \| 1.026 \| 0.147 \| 2.364 \| \| **ALS_10** \| 0.076 \| 1.233 \| 0.918 \| 0.722 \| 106.1 \| 8.79 \| 1.124 \| 0.167 \| 2.327 \| \| **ALS_11** \| 0.064 \| 1.190 \| 0.886 \| 0.804 \| 100.8 \| 9.12 \| 1.093 \| 0.140 \| 2.595 \| \| **ALS_12** \| 0.057 \| 1.257 \| 0.873 \| 0.790 \| 97.7 \| 9.18 \| 1.085 \| 0.151 \| 2.434 \| \| **ALS_13** \| 0.071 \| 1.204 \| 0.805 \| 0.743 \| 101.5 \| 8.90 \| 1.166 \| 0.169 \| 2.076 \| \| **ALS_14** \| 0.054 \| 1.277 \| 0.783 \| 0.725 \| 96.5 \| 9.26 \| 1.148 \| 0.140 \| 2.147 \| \| **ALS_15** \| 0.050 \| 1.246 \| 0.896 \| 0.648 \| 105.3 \| 8.89 \| 1.124 \| 0.139 \| 2.484 \| \| **ALS_16** \| 0.066 \| 1.257 \| 0.813 \| 0.716 \| 99.7 \| 9.18 \| 1.103 \| 0.152 \| 2.401 \| \| **ALS_17** \| 0.122 \| 1.150 \| 0.932 \| 0.838 \| 90.7 \| 9.70 \| 1.138 \| 0.151 \| 2.534 \| \| **ALS_18** \| 0.064 \| 1.189 \| 0.760 \| 0.695 \| 97.1 \| 9.59 \| 0.998 \| 0.170 \| 2.339 \| \| **ALS_19** \| 0.106 \| 1.180 \| 0.810 \| 0.810 \| 105.5 \| 8.48 \| 1.209 \| 0.135 \| 2.090 \| \| **ALS_20** \| 0.028 \| 1.240 \| 0.687 \| 0.617 \| 97.7 \| 9.00 \| 1.029 \| 0.172 \| 2.634 \| \| **ALS_21** \| 0.042 \| 1.385 \| 0.746 \| 0.564 \| 98.1 \| 9.17 \| 1.062 \| 0.164 \| 2.392 \| \| **ALS_22** \| 0.046 \| 1.272 \| 0.800 \| 0.687 \| 97.1 \| 9.21 \| 1.070 \| 0.155 \| 2.381 \| \| **ALS_23** \| 0.074 \| 1.111 \| 0.746 \| 0.719 \| 104.1 \| 8.81 \| 1.143 \| 0.158 \| 2.442 \| \| **ALS_24** \| 0.060 \| 1.470 \| 0.863 \| 0.690 \| 99.7 \| 9.10 \| 1.064 \| 0.180 \| 2.526 \| |  |  |  |  |  |  |  |  |  |  |  |  |  |
| --- | --- | --- | --- | --- | --- | --- | --- | --- | --- | --- | --- | --- | --- | --- | --- | --- | --- | --- | --- | --- | --- | --- | --- | --- | --- | --- | --- | --- | --- | --- | --- | --- | --- | --- | --- | --- | --- | --- | --- | --- | --- | --- | --- | --- | --- | --- | --- | --- | --- | --- | --- | --- | --- | --- | --- | --- | --- | --- | --- | --- | --- | --- | --- | --- | --- | --- | --- | --- | --- | --- | --- | --- | --- | --- | --- | --- | --- | --- | --- | --- | --- | --- | --- | --- | --- | --- | --- | --- | --- | --- | --- | --- | --- | --- | --- | --- | --- | --- | --- | --- | --- | --- | --- | --- | --- | --- | --- | --- | --- | --- | --- | --- | --- | --- | --- | --- | --- | --- | --- | --- | --- | --- | --- | --- | --- | --- | --- | --- | --- | --- | --- | --- | --- | --- | --- | --- | --- | --- | --- | --- | --- | --- | --- | --- | --- | --- | --- | --- | --- | --- | --- | --- | --- | --- | --- | --- | --- | --- | --- | --- | --- | --- | --- | --- | --- | --- | --- | --- | --- | --- | --- | --- | --- | --- | --- | --- | --- | --- | --- | --- | --- | --- | --- | --- | --- | --- | --- | --- | --- | --- | --- | --- | --- | --- | --- | --- | --- | --- | --- | --- | --- | --- | --- | --- | --- | --- | --- | --- | --- | --- | --- | --- | --- | --- | --- | --- | --- | --- | --- | --- | --- | --- | --- | --- | --- | --- | --- | --- | --- | --- | --- | --- | --- | --- | --- | --- | --- | --- | --- | --- | --- | --- | --- | --- | --- | --- | --- | --- | --- | --- | --- | --- | --- | --- | --- | --- | --- | --- | --- | --- | --- | --- | --- |

**Supplementary Table 2.** Imaging biomarkers values in the motor cortex for all 24 ALS patients.

| \| **Pt_ID**  **[ALS_#]** \| **GSH**  **/ Cr** \| **NAA**  **/ Cr** \| **Glx**  **/ Cr** \| **Ins**  **/ Cr** \| **T1ρ [ms]** \| **WBM [%]** \| **AD [mm^2^/ms]** \| **RD [mm^2^/ms]** \| **MD [mm^2^/ms]** \| **FA**  **[0-1]** \| **Volume [mm^3^]** \| \| --- \| --- \| --- \| --- \| --- \| --- \| --- \| --- \| --- \| --- \| --- \| --- \| \| **ALS_01** \| 0.100 \| 1.477 \| 0.809 \| 0.648 \| n.a. \| n.a. \| 1.314 \| 0.581 \| 0.825 \| 0.494 \| 1396 \| \| **ALS_02** \| n.a. \| n.a. \| n.a. \| n.a. \| 72.928 \| 11.28 \| 1.185 \| 0.515 \| 0.738 \| 0.506 \| 1821 \| \| **ALS_03** \| 0.118 \| 1.423 \| 0.840 \| 0.918 \| 79.751 \| 10.54 \| 1.330 \| 0.575 \| 0.827 \| 0.510 \| 1720 \| \| **ALS_04** \| 0.090 \| 1.158 \| 0.929 \| 1.110 \| 80.806 \| 10.58 \| 1.249 \| 0.557 \| 0.788 \| 0.500 \| 2144 \| \| **ALS_05** \| 0.101 \| 1.529 \| 1.057 \| 1.096 \| 78.062 \| 10.82 \| 1.292 \| 0.484 \| 0.753 \| 0.572 \| 2019 \| \| **ALS_06** \| 0.109 \| 1.786 \| 1.113 \| 1.068 \| 78.439 \| 10.69 \| 1.267 \| 0.522 \| 0.770 \| 0.538 \| 2007 \| \| **ALS_07** \| 0.083 \| 1.037 \| 0.818 \| 1.165 \| 80.888 \| 10.51 \| 1.330 \| 0.549 \| 0.809 \| 0.529 \| 1860 \| \| **ALS_08** \| 0.097 \| 1.669 \| 0.947 \| 0.715 \| 82.170 \| 10.32 \| 1.365 \| 0.495 \| 0.785 \| 0.590 \| 1844 \| \| **ALS_09** \| 0.093 \| 1.506 \| 0.974 \| 1.038 \| 79.332 \| 10.63 \| 1.252 \| 0.511 \| 0.758 \| 0.538 \| 2151 \| \| **ALS_10** \| 0.109 \| 1.595 \| 1.008 \| 0.859 \| 77.324 \| 10.95 \| 1.338 \| 0.541 \| 0.807 \| 0.547 \| 1837 \| \| **ALS_11** \| 0.056 \| 1.399 \| 0.933 \| 0.782 \| 75.186 \| 11.24 \| 1.307 \| 0.521 \| 0.783 \| 0.542 \| 1842 \| \| **ALS_12** \| 0.061 \| 1.599 \| 0.970 \| 0.944 \| 73.081 \| 11.40 \| 1.341 \| 0.452 \| 0.749 \| 0.614 \| 2015 \| \| **ALS_13** \| 0.075 \| 1.364 \| 0.878 \| 0.830 \| 77.615 \| 10.82 \| 1.349 \| 0.538 \| 0.808 \| 0.547 \| 1690 \| \| **ALS_14** \| 0.067 \| 1.363 \| 0.874 \| 0.765 \| 78.890 \| 10.75 \| 1.287 \| 0.561 \| 0.803 \| 0.509 \| 1971 \| \| **ALS_15** \| 0.086 \| 1.378 \| 0.930 \| 0.855 \| 81.123 \| 10.56 \| 1.357 \| 0.569 \| 0.831 \| 0.519 \| 1913 \| \| **ALS_16** \| 0.075 \| 1.441 \| 0.845 \| 0.737 \| 78.671 \| 10.76 \| 1.412 \| 0.547 \| 0.835 \| 0.567 \| 819 \| \| **ALS_17** \| 0.139 \| 1.426 \| 0.906 \| 0.921 \| 76.835 \| 10.81 \| 1.312 \| 0.568 \| 0.816 \| 0.511 \| 1872 \| \| **ALS_18** \| 0.076 \| 1.578 \| 0.875 \| 0.800 \| 78.157 \| 10.79 \| 1.374 \| 0.523 \| 0.807 \| 0.562 \| 2617 \| \| **ALS_19** \| 0.101 \| 1.460 \| 0.889 \| 0.929 \| 77.476 \| 10.85 \| 1.305 \| 0.512 \| 0.776 \| 0.561 \| 2216 \| \| **ALS_20** \| 0.038 \| 1.393 \| 0.766 \| 0.670 \| 77.609 \| 10.77 \| 1.283 \| 0.558 \| 0.800 \| 0.511 \| 1607 \| \| **ALS_21** \| 0.080 \| 1.551 \| 0.867 \| 0.704 \| 76.458 \| 11.04 \| 1.299 \| 0.509 \| 0.772 \| 0.557 \| 1946 \| \| **ALS_22** \| 0.073 \| 1.517 \| 0.913 \| 0.874 \| 78.528 \| 10.72 \| 1.360 \| 0.547 \| 0.818 \| 0.540 \| 2482 \| \| **ALS_23** \| 0.104 \| 1.409 \| 0.779 \| 0.772 \| 82.654 \| 10.27 \| 1.330 \| 0.535 \| 0.800 \| 0.538 \| 1831 \| \| **ALS_24** \| 0.065 \| 1.466 \| 0.853 \| 0.795 \| 77.021 \| 10.91 \| 1.173 \| 0.321 \| 0.605 \| 0.705 \| 3218 \| |  |  |  |  |  |  |  |  |  |  |  |  |  |
| --- | --- | --- | --- | --- | --- | --- | --- | --- | --- | --- | --- | --- | --- | --- | --- | --- | --- | --- | --- | --- | --- | --- | --- | --- | --- | --- | --- | --- | --- | --- | --- | --- | --- | --- | --- | --- | --- | --- | --- | --- | --- | --- | --- | --- | --- | --- | --- | --- | --- | --- | --- | --- | --- | --- | --- | --- | --- | --- | --- | --- | --- | --- | --- | --- | --- | --- | --- | --- | --- | --- | --- | --- | --- | --- | --- | --- | --- | --- | --- | --- | --- | --- | --- | --- | --- | --- | --- | --- | --- | --- | --- | --- | --- | --- | --- | --- | --- | --- | --- | --- | --- | --- | --- | --- | --- | --- | --- | --- | --- | --- | --- | --- | --- | --- | --- | --- | --- | --- | --- | --- | --- | --- | --- | --- | --- | --- | --- | --- | --- | --- | --- | --- | --- | --- | --- | --- | --- | --- | --- | --- | --- | --- | --- | --- | --- | --- | --- | --- | --- | --- | --- | --- | --- | --- | --- | --- | --- | --- | --- | --- | --- | --- | --- | --- | --- | --- | --- | --- | --- | --- | --- | --- | --- | --- | --- | --- | --- | --- | --- | --- | --- | --- | --- | --- | --- | --- | --- | --- | --- | --- | --- | --- | --- | --- | --- | --- | --- | --- | --- | --- | --- | --- | --- | --- | --- | --- | --- | --- | --- | --- | --- | --- | --- | --- | --- | --- | --- | --- | --- | --- | --- | --- | --- | --- | --- | --- | --- | --- | --- | --- | --- | --- | --- | --- | --- | --- | --- | --- | --- | --- | --- | --- | --- | --- | --- | --- | --- | --- | --- | --- | --- | --- | --- | --- | --- | --- | --- | --- | --- | --- | --- | --- | --- | --- | --- | --- | --- | --- | --- | --- | --- | --- | --- | --- | --- | --- | --- | --- | --- | --- | --- | --- | --- | --- | --- | --- | --- | --- | --- | --- | --- | --- | --- | --- | --- | --- | --- | --- | --- | --- | --- | --- | --- | --- | --- | --- | --- | --- | --- | --- | --- | --- | --- |

**Supplementary Table 3.** Imaging biomarkers values in the corticospinal tract for all 24 ALS patients.
